# Human interphase chromosomes: a review of available molecular cytogenetic technologies

**DOI:** 10.1186/1755-8166-3-1

**Published:** 2010-01-11

**Authors:** Svetlana G Vorsanova, Yuri B Yurov, Ivan Y Iourov

**Affiliations:** 1 Institute of Pediatrics and Children Surgery, Rosmedtechnologii, Moscow, 127412, Russia; 2 National Research Center of Mental Health, Russian Academy of Medical Sciences, Moscow 119152, Russia

## Abstract

Human karyotype is usually studied by classical cytogenetic (banding) techniques. To perform it, one has to obtain metaphase chromosomes of mitotic cells. This leads to the impossibility of analyzing all the cell types, to moderate cell scoring, and to the extrapolation of cytogenetic data retrieved from a couple of tens of mitotic cells to the whole organism, suggesting that all the remaining cells possess these genomes. However, this is far from being the case inasmuch as chromosome abnormalities can occur in any cell along ontogeny. Since somatic cells of eukaryotes are more likely to be in interphase, the solution of the problem concerning studying postmitotic cells and larger cell populations is interphase cytogenetics, which has become more or less applicable for specific biomedical tasks due to achievements in molecular cytogenetics (i.e. developments of fluorescence *in situ *hybridization -- FISH, and multicolor banding -- MCB). Numerous interphase molecular cytogenetic approaches are restricted to studying specific genomic loci (regions) being, however, useful for identification of chromosome abnormalities (aneuploidy, polyploidy, deletions, inversions, duplications, translocations). Moreover, these techniques are the unique possibility to establish biological role and patterns of nuclear genome organization at suprachromosomal level in a given cell. Here, it is to note that this issue is incompletely worked out due to technical limitations. Nonetheless, a number of state-of-the-art molecular cytogenetic techniques (i.e multicolor interphase FISH or interpahase chromosome-specific MCB) allow visualization of interphase chromosomes in their integrity at molecular resolutions. Thus, regardless numerous difficulties encountered during studying human interphase chromosomes, molecular cytogenetics does provide for high-resolution single-cell analysis of genome organization, structure and behavior at all stages of cell cycle.

## Introduction

Currently, it is estimated that no fewer than 1 million cytogenetic and molecular cytogenetic analyses are performed per year representing the standard of care in several fields of medicine and the routine clinical work-up for numerous patients suffering from congenital malformations, mental diseases, cancers, or reproductive problems [1]. Molecular cytogenetic techniques have been repeatedly proven effective in diagnostics and have been recognized as a valuable addition or even alternative to chromosomal banding [2-4]. Furthermore, contemporary basic biomedical research widely applies molecular cytogenetic technologies [5-7]. Browsing the most popular scientific resources would undoubtedly return several tens of thousands of articles, which mention at least one molecular cytogenetic technique (for more details refer to [3] and web page about multicolor fluorescence in situ hybridization at http://www.med.uni-jena.de/fish/mFISH/mFISHlit.htm managed by Dr. Thomas Liehr, Jena, Germany). Thus, one can be certain that it is hard to overestimate the role of molecular cytogenetics in current biomedicine.

There are two main advantages that molecular cytogenetics possesses: (i) the ability to provide either an on-chip scan of the whole genome at extremely high resolution or visualization of single peculiar genomic loci [4,6,8]; (ii) the capability to analyze genome organization, structure and behavior in single cells at the DNA (RNA) sequence level [7,9,10]. Both are continuously used for biomedical research and molecular diagnosis of chromosome abnormalities in humans [2-13]. The first advantage is appreciable when analyzing mixed DNA isolated from large amount of cells. Therefore, it is unsurprising that such approaches are rarely used for single-cell analysis [10,14]. The second advantage of molecular cytogenetic techniques is consistently emphasized [3,5-13], but is used more commonly for studying mitotic cells *via *analyzing metaphase chromosomes [3,7,10,12]. However, cells of eukaryotes are more likely to be in interphase. Therefore, during surveys of genome organization, structure and behavior, essential part of cellular life is usually fallen out of researchers' scope. As to molecular diagnosis of chromosome abnormalities, one can notice that interphase analysis is uncommonly applied, as well. The explanation of leaving interphase cytogenetics aside from diagnostics and research might be a suggested lack of reproducibility and low resolution. A brief look through studies of genome architecture in interphase nuclei [15-19] and somatic genomic variations [7,10,12,20-29] as well as developments in interphase cytogenetics [30-35] will reveal such assumptions unsupported and will show that laboratories elaborating such techniques are able to solve different practical and research tasks without major difficulties [3,7,12-35]. It seems thereby that preferences to use interphase molecular cytogenetic techniques suffer rather from "insufficient publicity" than from "technological underdevelopment".

Looking through the voluminous amount of reviews dedicated to molecular cytogenetics, we have found occasional descriptions of both technological and theoretical side of visualizing human chromosomes in interphase. Consequently, we were forced to conclude that undeservedly little attention is paid to interphase molecular cytogenetics in modern biomedical literature. Additionally, technical side of the application is even more rarely addressed. To fill this gap, we have attempted to give an overview of currently applied molecular cytogenetic techniques with a special emphasis on their technological abilities for studying human interphase chromosomes.

## Molecular cytogenetic techniques, their resolution and potential for single-cell analysis of interphase chromosomes

The overwhelming majority of molecular cytogenetic techniques are based on hybridization. There are currently two essential platforms available for developments in molecular cytogenetics: fluorescence in situ hybridization (FISH) including comparative genomic hybridization (CGH) [3,36] and peptide nucleic acid (PNA) probing for analysis of chromosomal DNA [37,38]. Alternatively, another technique uses primed in situ labelling (PRINS) reaction [37,38]. The resolution and level of excellence of all these techniques are established against cytogenetic banding analysis, which remains the golden standard in this instance [36].Single-cell molecular cytogenetic analysis can be performed either through analysis of metaphase plates or through analysis of interphase nuclei. Studying metaphase plates has been long described to be successful using several detection technologies (i.e. spectral karyotyping -- SKY or multicolor FISH -- MFISH) and different DNA probe sets (chromosome-enumeration/centromeric, site-specific, whole-painting, microdissected) [2,3,5-7,9-13,30,36,39-46]. In general, if modified, almost all these techniques can be applied to interphase cells, but this "transfer of technology" requires significant efforts [2,3,7,10,12,13,30-33,35,47]. Generally, all molecular cytogenetic assays that provide for visualization of genomic loci in an interphase nucleus are termed interphase FISH or I-FISH [35]. Table 1 gives an overview of molecular cytogenetic techniques that are used for metaphase and interphase analysis with special attention to the resolution and to the modifications for studying single cells. The impossibility of listing all known molecular cytogenetic approaches seems to be apparent, but even a short description of such techniques (Table 1) shows molecular cytogenetics able to perform high-resolution analysis of chromosomal structure and behavior at all stages of cell cycle, being, nevertheless, more frequently use to detect metaphase chromosome imbalances and rearrangements or to operate with total DNA for probing in CGH analysis [2-7,10-14,19-54]. Further, we attempt to review each aforementioned approach in context of applications to single-cell chromosomal analysis.

**Table 1:** Molecular cytogenetic techniques, their resolution and validity for single-cell analysis of interphase/metaphase chromosomes (for more details see text)

Approach	Resolution	MA*	IA**	SCA^^^	PVC^^^^	Refs
Cytogenetic banding analysis ("golden standard")	5-7 Mb	+	-	+	+	[1]

FISH/MFISH/SKY						

FISH/MFISH/SKY with centromeric probes	>0.3-1 Mb	+/-	+	+	+/-	[20-26,30,32,35,41,42,44,46]

FISH/MFISH/SKY with site-specific probes	~0.1-2 Mb	+/-	+/-	+/-	+/-	[45,47-50]

FISH/MFISH/SKY with whole-painting probes	>5-10 Mb	+	-	+	+	[2,3,5,6,13,36,39,40]

MCB						

Metaphase MCB	~2-5 Mb	+	-	+	+	[2,13,43,45]

ICS-MCB	~2-5 Mb	-	+	+	+	[23,24,26,28,29,31-35]

Fiber FISH	>2.3 (2-3) kb	na	na	+	+	[51,52]

Single-cell CGH						

Standard CGH	2-5 Mb	na	na	+	-	[53]

Array CGH	0.03-1 Mb	na	na	+	-	[14,54]

* -- analysis of metaphase chromosomes (MA - metaphase analysis); ** -- analysis of interphase chromosomes (IA - interphase analysis); ^^ ^-- possibility to perform single cell analysis (SCA); ^^^ ^-- possibility to visualize chromosomes or chromosomal loci (PVC - possibility to visualize chromosomal loci); na - not applicable;

### FISH

FISH offers numerous possibilities to study either the whole genome or specific genomic loci (regions) [2-7,10-13,36,39-41]. The probes mainly determine the resolution of molecular cytogenetic techniques [3]. Regardless molecular peculiarities and pattern of sequence modifications (i.e. LNA (locked-nucleic acid) or PNA probes, for more details see [3,13,37,38]), probes for molecular cytogenetic assays can be classified according to the pattern of detected DNA sequences. Such classification includes repetitive-sequence DNA (centromeric and telomeric), site-specific, whole chromosome painting (wcp) probes [3,55].

FISH, which paints repetitive genomic sequences, can be performed with either centromeric (chromosome enumeration or chromosome-specific) or telomeric DNA probes. Analysis of telomeres is an important area of biomedical research [56]. Usually, DNA or PNA probes possessing TTAGGG repetitive sequence motifs are used [3,56]. These assays are needed to cover large area of cancer and aging research (telomere biology), but seem to be poorly applicable for diagnosis [3]. I-FISH analysis using telomeric probes was only described in few nuclear organization studies [57]. Contrariwise, applications of I-FISH with centromeric DNA probes are an integral part of diagnostics in medical genetics, oncology and reproductive medicine [1-3,5,7,10,12,13,20-30,35-38,41,42,44,46,55,58-61]. Moreover, application of these probes has been long demonstrated to be extremely valuable for research in fields of chromosome biology studying genome organization (chromosomal and nuclear), evolution, behavior and variation in health and disease [2,3,7,10,12,13,20-30,35,41,42,44,55,62-67]. The popularity of these DNA probes is usually attributed to near 100% hybridization efficiency because of painting highly repetitive DNAs as well as to chromosome-specifity of centromeric human DNAs allowing analysis of individual homologous chromosome pairs in interphase [7,10,30,35]. Moreover, due to the extreme interindividual variations of pericentromeric heterochromatic DNA, such assays allow application of quantitative FISH (QFISH) that can be useful for solving numerous problems encountered during metaphase and interphase analysis of chromosomes [32,35,59]. The potential of related assays is poorly determined by its genomic resolution (Table 1), inasmuch as applications of centromeric DNA probes suggest the analysis of phenomena encompassing significantly larger genomic loci as to visualized ones [3,7,10]. As to interphase cytogenetics, I-FISH with chromosome-enumeration probes makes possible to detect numerical chromosome imbalances (aneuploidy and polyploidy) in vast cell populations [7,10,12,20-30,35,41,42,60]. In a limited amount of cases, similar approaches are applicable for metaphase cytogenetic analysis of chromosome abnormalities [58,63,64,68]. Numbers of signals for these probes are supposed to be identical to numbers of homologous chromosomes per interphase nucleus [3,7,10,20-30,33,35,41,42,46]. However, this is not always the case [7,10,23-33,35]. This is the main disadvantage of I-FISH with centromeric DNA probes, which is, however, successfully solved by means of FISH with site-specific DNA probes (locus-specific BAC probes or BAC-probe contigs) [13].

FISH using site-specific DNA probes (YACs, BACs, PACs, cosmids) is usually used to map chromosomal regions, within which a breakpoint is located [3,5,13,61]. Additionally, these probes can be used for diagnosing known microdeletion and microduplication syndromes [1,3,13,27], aneuploidy and recurrent chromosome abnormalities during preimplantation genetic diagnosis [48-50], prenatal diagnosis [3,13,47], oncocytogenetic analysis [1-3,5,13,36,27,50], and precision of copy number variations [8]. Being valuable approach for studying genomic loci smaller than 1 Mb, I-FISH with site-specific probes is frequently used for studying nuclear organization of genes and its impact on the transcriptional activity [16-18,69]. Nevertheless, relatively moderate hybridization efficiency (<70%) hinders the application of such approaches in numerous areas of biomedical research and diagnosis [7,10]. The latter does not concern a number of diagnostic FISH procedures applying these types of probes (for instance, in cases of routine oncohematological and tumor diagnostics). For diagnostic issues, such approaches has cut-offs between 92 and 98% [13].

FISH using wcp is a basis for MFISH (24-color FISH) and SKY [2,13,39,40]. These methods are valuable for cancer cytogenetics and, in some cases, for diagnosis of constitutional chromosome abnormalities [2,5,6,13,36]. For analysis of interphase chromosomes, MFISH/SKY is hardly applicable. Nevertheless, a study has visualized simultaneously all chromosomes in interphase nuclei of fibroblasts and prometaphase rosettes by 24-color MFISH [70]. Afterwards, such approaches have not been ever considered for related analysis. Two-to-five-color assays with wcp probes have been repeatedly used for molecular cytogenetic diagnosis of structural alterations to metaphase chromosomes [1-3,5-7,13,36,61] and investigation of genome organization in interphase nuclei [15,57,66,70-72]. I-FISH with wcp probes is too problematic to be competitive with other techniques of interphase molecular cytogenetic diagnosis [7,10,33].

By microdissection of chromosomal loci for obtaining a set of probes that produce multicolor pseudo-G-banding, a high-resolution molecular cytogenetic technique for analysis of metaphase chromosomes termed MCB (multicolor banding) was proposed [43]. The latter has been consistently shown to be applicable for the identification of structural chromosome abnormalities and genome organization [2,13,36,43,45,61,73]. A modification of this technique, called recently interphase chromosome-specific MCB (ICS-MCB) that generates MCB of a homologous chromosome pair on single nuclei, has been demonstrated effective for studying human interphase chromosome organization and variations (somatic genomic variations and chromosome instability in health and disease) [3,7,10,13,19,23,24,26-29,31,33-35,74,75]. Apart from impossibility to analyze simultaneously several homologous chromosome pairs and relative complexity of the analysis, ICS-MCB does not possess major limitations. Moreover, this is the unique way to obtain a view on the entire interphase chromosome in its integrity [23,33,35].

The highest molecular cytogenetic resolution is achieved by fiber FISH (~2.3 kb) [52,76]. This approach was originally designed for mapping cloned DNA fragments at high resolution. The latter was found useful for investigation of genomic organization (on metaphase chromosomes), stalled transcription and genomic rearrangements (including large deletions within gene sequences) [51,52,76]. Although this technique is based on obtaining DNA fibers from interphase nuclei, it cannot be attributed to I-FISH. Single-cell molecular cytogenetic analysis by fiber FISH (especially, analysis of large cell populations) is highly complicated.

### CGH

Since CGH compares quantitative differences between individual genomes, its applications are restricted to analysis of losses/gains of chromosomal (genomic) loci without direct visualization of chromosomes [4,77]. Array CGH can provide for a resolution up to nucleotide level, but still is poorly applicable for studying chromosomes of a cell. Nevertheless, several reports have demonstrated either standard CGH or array CGH on microdissected interphase nuclei to detect chromosome aberrations in single cells of preimplantation embryos [14,53,54]. Such approaches are applicable for unbalanced genomic rearrangements being useless for other areas of chromosome biology, which requires visualization of chromosomal DNA [10]. The potential of CGH-based single-cell analysis for molecular diagnosis and for surveys of somatic genomic variations remains to be estimated.

### PNA and PRINS

Both PNA and PRINS can be successfully applied for studying human chromosomes [3,7,10,13,37,38]. PNAs are suggested to have several advantages over conventional molecular cytogenetic DNA probes, which are the result of their smaller size [38]. Notwithstanding, poor availability does not allow researchers to evaluate *in situ *hybridization with PNA probes for either metaphase or interphase molecular cytogenetics. Moreover, these probes are usually restricted to studying centromeric and telomeric repetitive chromosomal DNA.

In contrast to FISH and CGH, PRINS is based on another biochemical process (polymerase reaction) [37]. This makes it useful for case-control studies of newly discovered phenomena to exclude hypothetical errors that might be produced by hybridization [24]. Usually, PRINS shows almost the same results as FISH. Therefore, there is no apparent interest to substitute FISH-based techniques by PRINS, especially taking into account its essential limitation: available probes are oligonucleotides for pericentromeric/heterochromatic and few euchromatic regions (poorly reproducible!) [3,7,24,37].

The key process of all the studies aimed to analyze interphase chromosomes is visualization. In other words, lacking of direct (microscopic) DNA visualization makes all such researches incomplete. This becomes even more evident for studying chromosome organization in single cells. As one can see, only FISH-based techniques offer possibilities to detect either whole chromosomes or specific genomic loci of extremely small size in single cells. Therefore, to perform a valid study of human interphase chromosomes, I-FISH protocols are to use. The next part of our review addresses areas of I-FISH applications as well as its advantages and limitations.

## I-FISH: advantages and limitations

I-FISH as all other FISH-based methods roughly requires three steps to be performed: (i) obtaining cells suspensions or performing another preparations of biopsies for the analysis; (ii) denaturation/hybridization; (iii) microscopic visual/digital analysis of hybridization results [13,35,78]. The first stage is not associated with any limitation of I-FISH, because any cell type of a human organism can be processed for such analyses [7,35,78]. This is considered the essential advantage of interphase molecular cytogenetic techniques in contrast to classical cytogenetics (analysis of metaphase chromosomes) -- the ability to analyze chromosomes in all the tissue (cell) types [3,7,13,20-36]. Classically, I-FISH was suggested to be limited to analyses of specific genomic loci [2,3,7,13]. However, some modifications such as ICS-MCB allow to get a view of interphase chromosomes in their integrity [23,24,26-29,31,33-35,74]. As mentioned before, ICS-MCB still have a limitation that is referred to the possibility of studying only one homologous chromosome pair per analysis (metaphase chromosomal analysis allows to visualize all chromosomes of a cell), being, however, the unique way to visualize the whole banded chromosome in a nucleus [23,33]. Denaturation and hybridization steps of I-FISH are performed identically to metaphase FISH-based approaches [13,35]. Therefore, no additional drawbacks can be attributed to these procedures during interphase molecular cytogenetic studies. Scoring of I-FISH results is usually performed *via *conventional visual analysis [35,79]. However, there are numerous possibilities to apply digital analysis for studying interphase chromosomes. These include, but are not restricted to, QFISH, analysis of signal co-localization (oncocytogenetic studies of gene fusions because of translocations in interphase nuclei), ICS-MCB (visualization of chromosomal structures), increasing of FISH result visibility, automatic signal detection [79]. Furthermore, digital analysis is a need for multicolor FISH-based assays (SKY, MFISH, multiprobe interpahse FISH or mFISH), which are usually applied to increase the potential of FISH applications through simultaneous analysis of multiple targets [2,3,12,13,20-30,35,36,80]. Combining several aforementioned techniques mFISH with 2-5 probes (colors) per assay, QFISH and ICS-MCB) has become a basis for an integrated approach proven to be of highest efficiency for molecular diagnosis and genome/chromosome researches at supramolecular level in interphase [7,10,12,13,20-30,32,33,35,41,60,78,81]. The type of FISH result evaluation (i.e. visual or digital) is determined by the type of assay or, more precisely, by features of DNA probes (amount of probes per reaction and DNA sequence affinity) and detection. Therefore, to get an overview it is to subdivide I-FISH techniques this way. Table 2 gives such overview.

**Table 2:** Overview of I-FISH techniques

Technique	Brief description	Advantages	Limitations	Refs
I-FISH with centromeric probes	I-FISH on interpahse nuclei painting pericentromeric (heterochromatic) regions	High hybridization efficiency, chromosome specifity (apart few chromosomes)	Signal associations, impossible to analyze chromosomes 5,13, 14, 19, 21, 22; heteromorphisms	[7,10,20-22,30,35,41,58-60,81,82]

I-FISH with site-specific probes	I-FISH painting specific euchromatic genomic loci	Small specific genomic loci are visualized	Low hybridization efficiency, numerous artifacts	[8,13,28,42,47-50,69]

I-FISH with wcp	I-FISH painting chromosome territories	Identification of nuclear chromosome territories	Chromosome territories are ambiguous, no additional information	[57,70-72,93]

mFISH	Multicolor I-FISH with >2 probes labeled by different fluorochromes/ligands	Analysis of several targeted genomic loci	Difficulty to distinguish between artifacts and aneuploidy/polyploidy	[7,10,20-22,30,35]

mFISH/QFISH	mFISH + QFISH digitalization of FISH signals	Distinguishes between FISH artifacts and aneuploidy (polyploidy)	Same as mFISH	[7,10,24-29,32,35]

MFISH	Simultaneous visualization of the complete set of chromosomes in an interphase nucleus	All chromosome territories are simultaneously seen	Exceedingly sophisticated analysis; data poorly interpretable	[70,93]

ICS-MCB	Chromosome-specific MCB generated on interphase nuclei	Visualization of whole banded interphase chromosomes in their integrity	A pair of homologous chromosomes is studied per assay; relative complexity of the analysis	[7,10,13,19,23,24,26-29,31,33-35,74,75]

### I-FISH with centromeric probes

**Figure 1:**
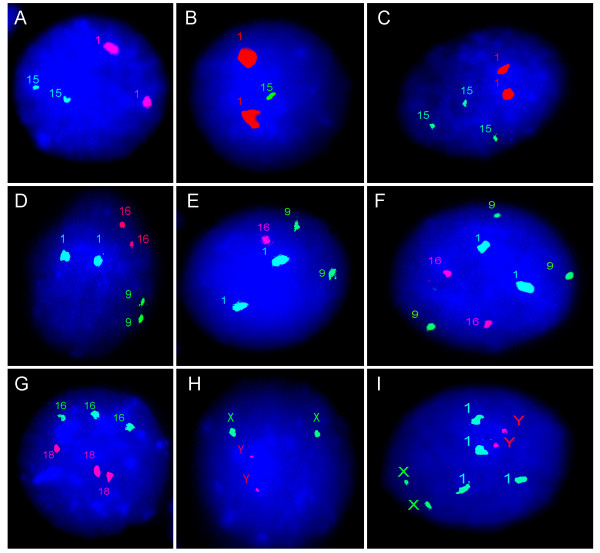
**Two- and three-color I-FISH with centromeric DNA probes**. **(A) **normal diploid nucleus with two signals for chromosome 1 and chromosome 15; **(B) **monosomic nucleus with two signals for chromosome 1 and one signal for chromosome 15; **(C) **trisomic nucleus with two signals for chromosome 1 and three signals for chromosome 15; **(D) **normal diploid nucleus with two signals for chromosome 1, chromosome 9 and chromosome 16; **(E) **monosomic nucleus with two signals for chromosome 1 and chrosmome 9 and one signal for chromosome 16; **(F) **trisomic nucleus with two signals for chromosome 1 and chromosome 16 and three signals for chromosome 9; **(G) **triploid nucleus with three signals for chromosome 16 and chromosome 18; **(H) **tetraploid nucleus with two signals for chromosome X and chromosome Y; **(I) **tetraploid nucleus with two signals for chromosome X and chromosome Y, and four signals for chromosome 1.

I-FISH with centromeric probes is highly applicable for different areas of biomedical research and diagnosis [7,10,13,20-22,30,35,41,58-60,81,82]. The most frequent application of the method is the identification of numerical chromosome abnormalities (aneuploidy and polyploidy) in interphase nuclei (Figure 1). The latter is required for pre-/postnatal diagnosis, cancer diagnosis/prognosis, somatic genomic variation surveys [7,10,20-22,30,35,82]. As one can see from table 2, near 100% hybridization efficiency of centromeric DNA probes [7,10,30,35] and chromosome-specific DNA sequences forming pericentromeric/heterochromatic chromosomal regions (apart from shared alphoid DNA of chromosomes 5 and 19, 13 and 21, 14 and 22) [30,35,41,42,44,83] are the essential source of advantages that this technique possesses. Heteromorphisms of pericentromeric DNAs can produce the lack of a signal leading, thereby, to impossibility of the I-FISH assay application. Fortunately, such extreme heteromorphisms (centromeric DNA variations) are rare in the general population [32,35,59,84-86].

### I-FISH with site-specific probes

**Figure 2:**
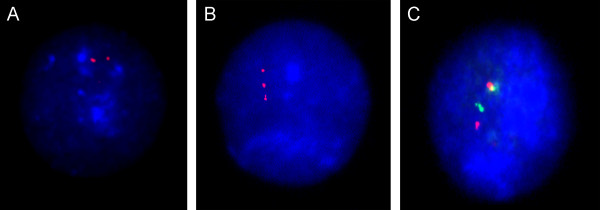
**I-FISH with site-specific DNA probes**. **(A) **normal diploid nucleus with two signals for chromosome 21; **(B) **trisomic nucleus with three signals for chromosome 21; **(C) **interphase nucleus exhibiting co-localization of *ABL *and *BCR *genes probably due to t(9;22)/Philadelphia chromosome.

**Figure 3:**
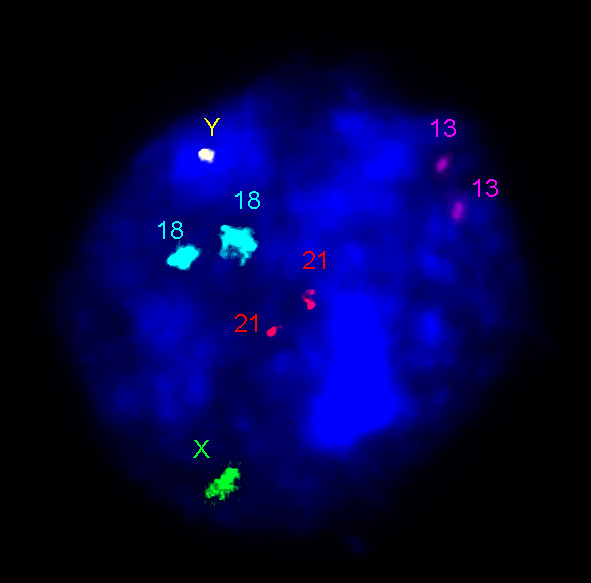
**Five-color I-FISH (mFISH) with DNA probes for chromosomes 18, X and Y (centromeric probes) as well as 13 and 21 (site-specific probes)**. a presumably normal (diploid) male nucleus isolated from the adult human brain.

Interphase molecular cytogenetic studies by I-FISH with site-specific probes are commonly applied in preimplantation, prenatal and postnatal diagnosis as well as in cancer cytogenetics (Figure 2) [2,3,13,36,47-50]. Although repeatedly noted to be of significant importance for detecting gene fusions resulting from interchromosomal translocations (cancer biomarkers) [49,87-89] and to be useful for preimplantation diagnosis [48-50], such I-FISH modifications has considerable disadvantages. Firstly, hybridization efficiency of site-specific probes is usually between 40 and 70% [7,10]. This has the potential to produce false-positive or false-negative data [7,28]. Additionally, it requires to use probes for "well-characterized" genomic DNA sequences (i.e. mapped oncogenes, genes/genomic loci within microdeletion or microduplication regions) [3]. Therefore, it is not surprising that there are only few approaches using these DNA probes that are performed to detect well-known chromosomal rearrangements in cancer cells [87-89] and, more rarely, deletions/duplications in clinical populations [1,3,8,50,90-92]. However, FISH using site-specific probes is almost the unique way to visualize DNA sequences smaller than 1 Mb in interphase nuclei. Simultaneous use of centromeric and site-specific probes in an mFISH assay (Figure 3) is sometimes useful for diagnostics and survey of intercellular (somatic) genomic variations [7,20,28,46,48].

### I-FISH with wcp

**Figure 4:**
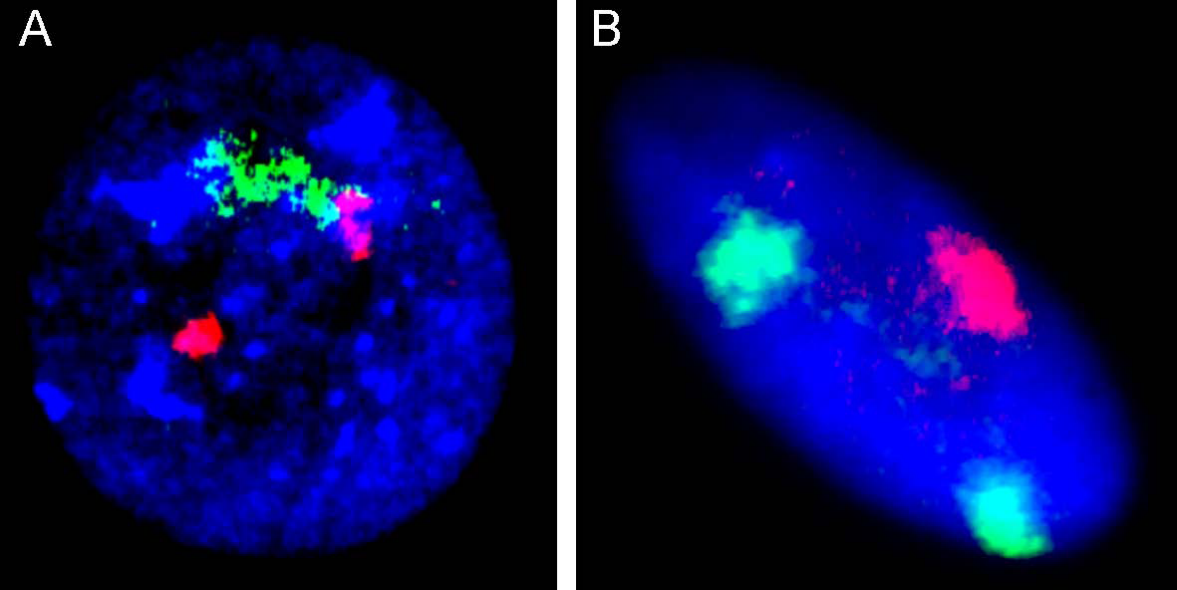
**I-FISH with two wcp for chromosomes 7 and 21**. **(A) **ambiguous chromosome territories provide information neither about number of chromosomes nor about structure of chromosomes (chromosome 7 -- green signal; chromosome 21 -- red signals), whereas this individual presented with regular unbalanced t(7;21); more details are given in Vorsanova et al. 2008 [64]; **(B) **chromosome territories in an interphase nucleus of a cell isolated from the ataxia-telangiectasia brain (chromosome 7 -- green signals; chromosome 14 -- red signal); note the impossibility to identify number of chromosomes 14.

It is generally recognized that FISH chromosomal painting using wcp is completely useless for identification of number and structure of interphase chromosomes (Figure 4) [3,7,10,13,33,35,80]. However, basic research of chromosome architecture in interphase is usually performed using I-FISH with wcp. These probes allows to visualize chromosome territories and their positioning relative to nuclear compartments (Figure 4B) [57,70-72,85,93]. For the last two decades, I-FISH-wcp approaches were almost the unique way to study genomic organization in interphase [72]. Some studies proposed to use the complete wcp set in an interphase MFISH reaction [70,93]. Nonetheless, these techniques are all limited in their abilities to paint chromosome territories (volumes) only (Table 2) [33].

### ICS-MCB

**Figure 5:**
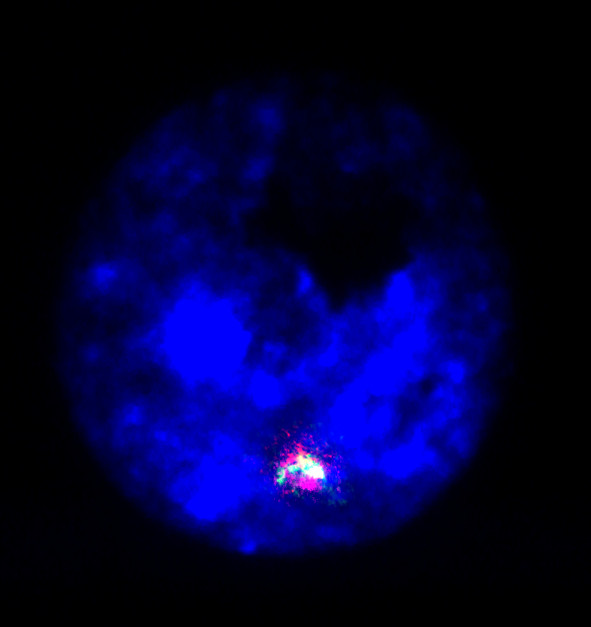
**ICS-MCB with chromosome 21-specific probe**. Monosomy (loss) of chromosome 21 in a nucleus isolated from the Alzheimer's disease brain.

To visualize a homologous pair of interphase chromosomes in their integrity, one has to generate MCB. Interphase banded chromosomes appear as metaphase ones when ICS-MCB is applied. Therefore, this I-FISH approach solves the long-standing limitation of cytogenetics that refers to obtaining metaphase chromosomes [23,31-35]. Figure 5 gives an example of aneuploidy detection in an intephase nucleus isolated from the Alzheimer's disease brain [28]. ICS-MCB can be widely applied for basic research of somatic genomic variations, chromosome structural and functional organization in interphase, supramolecular disease mechanisms [3,7,10,12,13,19,23,24,26-29,31,33-36,73-75,79-81]. Apparently, the sole disadvantage of this technique is the impossibility to analyze more than one homologous chromosome pair at once [23,33].

**Figure 6:**
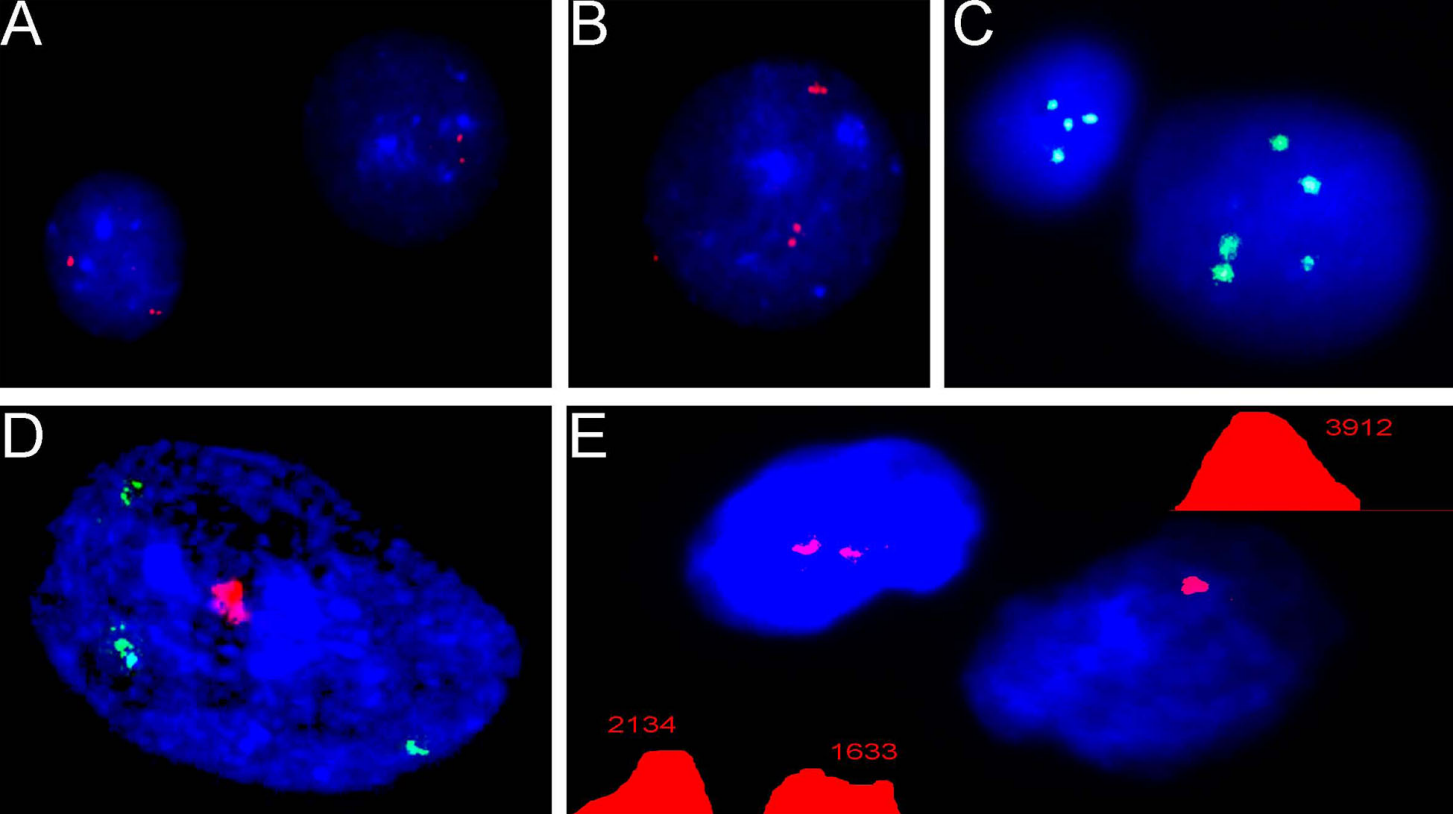
**Problems of I-FISH with centromeric/site-specific DNA probes**. **(A) **and **(B) **replication of specific genomic loci (LSI21 probe) -- some nuclei exhibit replicated signals, whereas in some nuclei it is not apparent; note the distance between signals can be more than a diameter of a signal; **(C) **asynchronous replication of a signal (DXZ1) in case of tetrasomy of chromosome X; note the difficulty to make a definitive conclusion about number of signals in the right nucleus; **(D) **Two-color FISH with centromeric/site-specific DNA probes for chromosome 1 shows chromosomal associations in a nucleus isolated from the adult human brain; note the impossibility to identify number of chromosomes; **(E) **QFISH demonstrating an association of centromeric regions of homologous chromosomes 9, but not a monosomy or chromosome loss (for more details see [32]).

There are several general problems that surround I-FISH application. As we have already mentioned, differences of hybridization efficiency complicate simultaneous applications of different probe sets [7]. For instance, signals of site-specific probes can be missed because of high brightness of wcp or centromeric probe signals. Here, the most apparent solution is ICS-MCB application [33,35]. However, some interphase protocols, mostly associated with molecular oncocytogenetics, are proven to be valid for diagnostic purposes [1,13,36,87,88]. DNA replication during S phase of cell cycle is another major problem of I-FISH applications [7,47]. Despite of recommendations concerning this type of I-FISH artifacts in the available literature, FISH analysis can be hindered by replicative signal appearance. This is mainly related to site-specific DNA probes, being, however, noticed during I-FISH with centromeric probes, as well [7,10,22,35,47] (Figure 6A-C). Additional source of numerous artifacts that can be considered as false-positive chromosome abnormalities in interphase is nuclear organization. In this context, the most problematic pattern of chromosome arrangement in the nucleus is related to chromosomal loci associations [94,95]. This significantly affects I-FISH results becoming even more important taking into account that numerous cell types are prone to exhibit intranuclear associations/pairing of genomic loci (Figure 6D) [20,32,35,95]. Regardless frequent occurrence of related difficulties, the problem is easily solved by QFISH (Figure 6E) [23,24,28,32,35,95].

**Figure 7:**
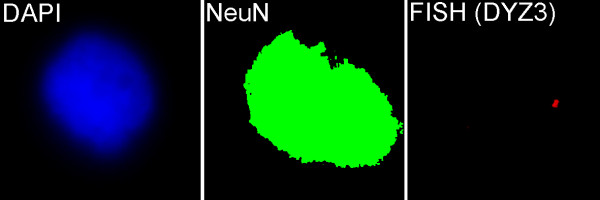
**Immuno-FISH**. I-FISH using centromeric probe for chromosome Y (DYZ3) with immunostaining by NeuN (neuron-specific antibody) performed for the analysis of cells isolated from the human brain.

Finishing the list of interphase FISH-based techniques, it is to mention Immuno-FISH. This method combines immunohistochemical detection of proteins and FISH for visualization of DNA (RNA) targets [96-98]. Immuno-FISH is found applicable in cancer research/diagnosis (simultaneous immunophenotyping and single-cell genetic analysis), studies of chromosome structure and organization, transplantation research, and identification of supramolecular disease mechanisms [28,29,96-100]. Figure 7 demonstrates Immuno-FISH used for studying interphase chromosomes in neuronal cells of the adult human brain [28,29].

After listing the most known methods of interphase molecular cytogenetics, it is to focus on their specific applications. Currently, there are there main biomedical areas requiring the use of I-FISH: analysis of intranuclear chromosome (genome) organization; identification of somatic (intercellular and intertissular) genomic variations; molecular cytogenetic diagnosis. Below, a brief description of these applications is given.

## Genome organization in interphase

Spatial chromosome organization in interphase has been repeatedly shown to be a driving force for numerous crucial intracellular processes. It is suggested that specific arrangement of interphase chromosomes is likely to associate with genome activity, normal/abnormal cell division, chromosome rearrangements occurring during meiosis and mitosis [7,15-17,69,19-72,75,93,100,101]. To get an integrated view of genome organization in interphase, numerous approaches should be applied. The leading role in these studies is played by I-FISH [7,80,72,93]. There could be several applications of I-FISH approaches for interphase chromosome analysis on this occasion: (i) identification of chromosome positioning and its relation to other nuclear compartments (nucleolus, Cajal bodies, nuclear speckles etc.) -- I-FISH with wcp, interphase MFISH or ICS-MCB [19,31,23,33,35,34,70-72,74,93]; (ii) studying correlation between positioning of specific genomic loci in relation to each other (i.e. association of whole chromosomes or their regions) and their behavior (transcriptional/replicative activity) for elucidating functional meaning of nuclear organization and its driving forces -- I-FISH with centromeric, site-specific and wcp, mFISH/QIFSH or ICS-MCB [7,19,31,23,33,35,32,34,57,66,69,72,74,75,93-95,100]; (iii) analysis of chromosome behavior in relation to genome, epigenome and proteome changes for delineation of possible consequences of specific interphase chromosome architecture (i.e. occurring of somatic chromosomal mutations in cancers) -- I-FISH with centromeric, site-specific and wcp, mFISH/QIFSH, ICS-MCB and Immuno-FISH [7,15-19,34,69,71,72,74,75,93-95,100,101]. Additional complication of I-FISH analysis of spatial chromosome organization is associated with structural preservation of nuclei. It is to note, that some researchers report about dependence of fixation type on I-FISH results [72,93], whereas others do not [71]. Regardless these debates, an alternative for I-FISH spatial genome analysis could be a suspension FISH (S-FISH) technique [102]. The advantage of this approach is related to possibility of studying three-dimensional (3D) preserved nuclei from any human tissue, whereas other 3D preservation techniques require specific conditions of cell cultivation. The latter makes I-FISH to lose its main advantage. Together, it is to conclude that comprehensive description of functional significance of nuclear organization requires application of almost all known interphase molecular cytogenetic techniques.

## Somatic genomic variations

During the last half decade, genomic variations -- a source of human healthy and pathological diversity -- have become a major focus of current biomedical research. Being involved in evolutionary and disease pathways, variations of the human genome are considered the main target of researches aimed to uncover disease mechanisms and species origins [103]. Soon after description of high rate of interindividual genomic diversification, it has been hypothesized that related processes-- somatic genomic variations -- lie at the origin of intercellular genomic differences. Moreover, somatic variability of cellular genomes was proposed as a mechanism for complex human diseases [7,10,12]. The latter has been partially confirmed by high-resolution interphase molecular cytogenetic (molecular neurocytogenetic) studies of neurological and psychiatric diseases [7,20-29]. The growing evidence for contribution of somatic genomic variations to the key physiological processes has been used for further hypothesizing about the emerging role of cell-to-cell genome variability in normal/abnormal human intrauterine development (including exogenous effects), cancerization, tissue-specific pathology (i.e. targeted neurodegeneration), sex differences in complex diseases, responses to molecular therapy of debilating neurological disorders [21,22,24,28,29,104-109]. Altogether, this forms a basis for forthcoming researches in the field of single-cell biology. All these achievements were the result of numerous developments in interphase molecular cytogenetics. To prove it, we would like to refer to determination of stochastic (sporadic or background) aneuploidy level in human tissues (Table 3) [20-24,59,28,29,35,81,109-112]. Looking through these data, it is hard to avoid the conclusion that aneuploidy rates become more reasonable if high-resolution I-FISH approaches are applied. Additionally, interindividual genomic variations can be detected in interphase by a parent-of-origin-determination FISH (pod-FISH) technique [113]. Together, I-FISH can be proposed as a required addition for studying genomic variations at microscopic and submicroscopic levels.

**Table 3:** Data on sporadic aneuploidy in different human tissues (presumably normal) depending on techniques used for the evaluation

Tissue	Technique	Aneuploidy rate	Refs
Ovarian tissues	I-FISH with site-specific probes	Statistically significant proportion of aneuploid cells (trisomy 21)	[109]

Chorionic villi	mFISH/QFISH ICS-MCB	~24% (~1% per chromosome)	[21,24,35]

Fetal human brain	mFISH/QFISH ICS-MCB	~30% (~1.5% per chromosome) ~35% + confined mosaicism	[22,24,35]

Blood	I-FISH with centromeric probes	Chromosome X: 1.5%-2.5% and 4.5%-5%*; Autosomes: 1.2% and 1%	[110]
	
	mFISH/QFISH	Chromosome X: 1.11%; Autosomes: 0,73%	[25]

Skin	mFISH	2,2% and 4,4%* (whole genome -- over 50%)	[111]

Liver	mFISH	~3% (whole genome -- over 50%)	[112]

Adult human brain	mFISH/QFISH ICS-MCB	~10% (~0.5% per chromosome)	[20,22,23,26,28,29]

* - in young and old individuals, respectively;

## Molecular cytogenetic diagnosis

Molecular cytogenetic identification of chromosomal aberrations by I-FISH has been already mentioned in this review. Here, we would like to make some additional comments related to more specific problems of medical cytogenetics and to show again that studying chromosomes in interphase nuclei has profound effects on molecular cancer and prenatal diagnosis [114,115]. It is obvious that it is almost impossible to refer all the studies that used I-FISH. Here, we have preferred to describe several difficulties encountered during I-FISH introduction and usage for diagnostic purposes. Newly introduced interphase techniques (i.e. ICS-MCB) were used for research purposes only and, therefore, have not been tested for diagnostic validity. Despite of limiting practical application of these I-FISH protocols, related drawbacks can be easily eliminated by forthcoming studies. Another problem comes from the diagnosis of chromosomal mosaicism. There do not exist commonly accepted guidelines or criteria for mosaicism definition [7,10,35]. Regardless some attempts (for details see [35]), there is still no consensus concerning this topic. The solution would be a large-scale study aimed to uncover somatic genomic variations in unaffected human tissues. Hopefully, similar studies have been already launched [20-24,59,28,29,35,81]. Finally, there are still no data or recommendations concerning correlation between metaphase and interphase diagnostic analysis of the same individual. In other words, it is still poorly understood what data is more valid. The structural point of view insists that metaphase analysis of chromosomes is more precise. From the other hand, mosaics require large cell populations to be analyzed. It becomes even more difficult to solve this problem when cases of complex, hidden (cryptic) or dynamic mosaicism are attempted to be described. Metaphase analysis in these case is indispensable for thorough definition of all cell lines, because simple I-FISH analyses are unable to precise a percentage of each cell line [116,117]. Moreover, some studies require additional data to obtain, i.e. parental origin of chromosomes or some epigenetic features for more thorough confirmational or exclusive diagnosis. To get this opportunity, it is to apply QFISH [32] or pod-FISH [112].

It is widely accepted that molecular cytogenetic diagnosis should be performed using a panel of techniques [1-10]. It could be either a combination of molecular cytogenetic techniques that use different platforms (i.e. FISH+CGH) or consecutive metaphase and interphase FISH analyses in cases of complex mosaics or balanced structural chromosome abnormalities. Thus, regardless significant developments in the field of molecular interphase cytogenetics, I-FISH techniques remain an addition to metaphase cytogenetics or whole genome screening approaches based on array CGH. The exception is few targeted assays for identification of known caner-associated translocations in interphase and preimplantation genetic diagnosis. Consequently, I-FISH should be more thoroughly analyzed in terms of the diagnostic potential to take a well-deserved place among genetic testing procedures.

## Conclusions and future directions

Structural and behavioral properties of human interphase chromosomes in different tissue/cell types in health and disease remain largely unknown. To date, only fragmentary data on distantly related areas of interphase chromosome biology are available without an integral view of chromosome behavior and arrangement along cell cycle. An overview of molecular cytogenetic techniques for visualizing chromosomes in interphase evidences that a strong technological basis does exist for high-resolution analyses of chromosomes of almost all human tissues. Three main directions of I-FISH application has been advanced by developments in interphase molecular cytogenetics which has provided for possibilities to define functional consequences of spatiotemporal chromosome arrangement in the nuclei, to elucidate the role of such immense intercellular genomic diversity (somatic genomic variations), to propose new diagnostic solutions for medical genetics and oncology. I-FISH is the unique way to study variations and behavior of the genome in all the cell types of human organism, at all stages of cell cycle and at molecular and supramolecular resolutions. Thus, developments in interphase molecular cytogenetics open numerous prospects for genetics, cellular and molecular biology, genomic/molecular medicine. Taking into account data on technological aspects of studying human interphase chromosomes, we conclude that this biomedical direction has the potential to provide revolutionary solutions for basic and applied biomedical research in fields of human genetics and cell biology. This would be undoubtedly the result of combination of interphase molecular cytogenetic techniques (i.e. mFISH, QFISH, ICS-MCB, S-FISH, pod-FISH, Immuno-FISH etc), which has already given rise to several discoveries in current biomedicine.

## Competing interests

The authors declare that they have no competing interests.

## Authors' contributions

SGV, YBY and IYI wrote the manuscript. All authors read and approved the final manuscript.
